# First Draft Genome Sequences of Two *Bartonella tribocorum* Strains from Laos and Cambodia

**DOI:** 10.1128/genomeA.01435-17

**Published:** 2018-01-11

**Authors:** Linda Hadjadj, Tawisa Jiyipong, Fadi Bittar, Serge Morand, Jean-Marc Rolain

**Affiliations:** a Unité de Recherche sur les Maladies Infectieuses et Tropicales Émergentes (URMITE), UMR CNRS, IHU Méditerranée Infection, Faculté de Médecine et de Pharmacie, Aix-Marseille Université, Marseille, France; b Institut des Sciences de l’Evolution, UMR CNRS–IRD, Université Montpellier, Montpellier, France; c ASTRE, CIRAD, Faculty of Veterinary Technology, Kasetsart University, Bangkok, Thailand

## Abstract

*Bartonella tribocorum* is a Gram-negative bacterium known to infect animals, and rodents in particular, throughout the world. In this report, we present the draft genome sequences of two strains of *B. tribocorum* isolated from the blood of a rodent in Laos and a shrew in Cambodia.

## GENOME ANNOUNCEMENT

*Bartonella* is a genus of Gram-negative bacteria. As facultative intracellular parasites, *Bartonella* species can infect humans and wild and domestic animals. *B. tribocorum* was first isolated from the blood of wild rats in France (1). Infections caused by this species in animals, and specifically in rodents, have been reported worldwide (2–5). Recently, it was also considered to be a zoonotic species that could cause undifferentiated chronic illness in humans following tick bites (6).

In 2011, a large series of blood samples (*n* = 1,341) from rodents and shrews trapped in Cambodia, Laos, and Thailand was collected (2). The presence of *Bartonella* spp. was screened by quantitative PCR (qPCR) (7). Positive samples were also tested by a culture method on Columbia agar supplemented with 5% sheep’s blood and incubated at 37°C in 5% CO_2_ for up to 4 weeks. In this study, we sequenced the genome of two *B. tribocorum* strains, L103 (CSUR P2060) and C635 (CSUR P2059), suspected to be new species. Strains L103 and C635 exhibited similarities with *B. tribocorum* strain BM1374166 of 96% and 96.24%, respectively, according to the partial *rpoB* gene (locus tags CER18_05755 and CEV08_05060, respectively), and 96.75% and 96.16%, respectively, according to the partial *gltA* gene (locus tags CER18_05610 and CEV08_03755, respectively). This is just over the cutoffs of 95.4% for the partial *rpoB* gene and 96% for partial *gltA* gene used to discriminate species of *Bartonella* (8). Strain L103 was isolated from a rodent, *Mus cookii*, trapped in the province of Luang Prabang, Laos, while strain C635 was isolated from a shrew, *Suncus murinus*, trapped in the province of Sihanouk, Cambodia.

Genomic DNA (gDNA) of *B. tribocorum* strains L103 and C635 were sequenced on a MiSeq sequencer (Illumina, Inc., San Diego, CA, USA) using the paired-end strategy. Raw reads were assembled with A5-miseq software (9). Genome and subsystem-based annotations were performed by Rapid Annotation using Subsystem Technology (RAST) (10, 11). tRNA gene detection was performed using the tRNAscan-SE 2.0 tool (12), whereas rRNA genes were predicted using RNAmmer (13). Plasmid presence was checked by PlasmidFinder software (14).

After assembly, the two genomes were composed of 99 scaffolds. For strains L103 and C635, the total sizes were 2,193,610 bp, with G+C contents of 38.4%, and 2,098,038 bp, with G+C contents of 38.0%, respectively. The draft genomes of *B. tribocorum* strains L103 and C635 contained 2,160 and 2,113 coding sequences, respectively, with 3 rRNAs and 40 tRNAs each. The RAST annotation assigned these genes to 291 and 290 subsystems, respectively, for strains L103 and C635, with a maximum number of genes associated with protein metabolism (17.66% and 17.53%, respectively), followed by amino acids and derivative metabolism (10.30% and 11.61%, respectively), and cofactors, vitamins, prosthetic groups, and pigment subsystems (8.50% and 9.09%, respectively). No studied strains carried a plasmid.

To our knowledge, these are the first draft genome sequences of *B. tribocorum* in Laos and also the first in Cambodia.

### Accession number(s).

These draft genome sequences have been deposited in NCBI GenBank under the sequence accession numbers NJGE00000000 and NJPP00000000 for strains L103 and C635, respectively.
